# West Nile Virus Occurrence and Ecological Niche Modeling in Wild Bird Species and Mosquito Vectors: An Active Surveillance Program in the Peloponnese Region of Greece

**DOI:** 10.3390/microorganisms10071328

**Published:** 2022-06-30

**Authors:** Marina Sofia, Alexios Giannakopoulos, Ioannis A. Giantsis, Antonia Touloudi, Periklis Birtsas, Kontantinos Papageorgiou, Zoi Athanasakopoulou, Dimitris C. Chatzopoulos, Georgia Vrioni, Dimitrios Galamatis, Vassilis Diamantopoulos, Spyridoula Mpellou, Evanthia Petridou, Spyridon K. Kritas, Matina Palli, Giorgos Georgakopoulos, Vassiliki Spyrou, Athanassios Tsakris, Alexandra Chaskopoulou, Charalambos Billinis

**Affiliations:** 1 Faculty of Veterinary Science, University of Thessaly, 43100 Karditsa, Greece; msofia@uth.gr (M.S.); algiannak@uth.gr (A.G.); atoul@uth.gr (A.T.); zathanas@uth.gr (Z.A.); 2 European Biological Control Laboratory, USDA-ARS—U.S. Department of Agriculture Agricultural Research Service, 57001 Thessaloniki, Greece; giantsisg@gmail.com (I.A.G.); achaskopoulou@ars-ebcl.org (A.C.); 3 Faculty of Forestry, Wood Science and Design, 43100 Karditsa, Greece; birtsas@uth.gr; 4 Faculty of Public and One Health, University of Thessaly, 43100 Karditsa, Greece; pgkostas@yahoo.gr (K.P.); dchatzopoulos@uth.gr (D.C.C.); 5 Faculty of Veterinary Medicine, Aristotle University of Thessaloniki, 54124 Thessaloniki, Greece; epetri@vet.auth.gr (E.P.); skritas@vet.auth.gr (S.K.K.); 6 Department of Microbiology, Medical School, National and Kapodistrian University of Athens, 15772 Athens, Greece; gvrioni@med.uoa.gr (G.V.); atsakris@med.uoa.gr (A.T.); 7 Hellenic Agricultural Organization DIMITRA (ELGO DIMITRA), 54248 Thessaloniki, Greece; galamatis@elog.gr; 8 Directorate of Public Health, Prefecture of Peloponnese, 22131 Tripoli, Greece; diamantopoulosv@yahoo.com; 9 Bioefarmoges Eleftheriou LP-Integrated Mosquito Control, 19007 Marathon, Greece; smpellou@bioefar-moges.com; 10 Wildlife Protection & Rehabilitation Center, 24400 Gargalianoi, Greece; keppazgr@gmail.com (M.P.); ggeogr@gmail.com (G.G.); 11 Faculty of Animal Science, University of Thessaly, 41110 Larissa, Greece; vassilikispyrou@uth.gr

**Keywords:** West Nile Virus, Peloponnese Region, active surveillance system, wild birds, mosquito, spatial analysis, vector control

## Abstract

West Nile Virus (WNV) is maintained in nature in a bird-mosquito cycle and human infections follow a seasonal pattern, favored by climatic conditions. Peloponnese Region, located in Southern Greece, initiated an active WNV surveillance program to protect public health during 2019–2020. The project included monitoring of avian hosts and mosquito vectors, while sampling locations were prioritized after consideration of WNV circulation in birds, mosquitos and humans during previous seasons. Biological materials were collected from 493 wild birds of 25 species and 678 mosquito pools, which were molecularly screened for WNV presence. In this case, 14 environmental variables were associated with WNV detection in wild birds and mosquitos by using two separate MaxEnt models. Viral RNA was not detected in the target species during 2019, although in 2020, it was reported on 46 wild birds of ten species and 22 mosquito pools (*Culex pipiens* and *Aedes albopictus*). Altitude and land uses were significant predictors for both models and in fact, suitable conditions for virus occurrence were identified in low altitude zones. Bird- and mosquito-based surveillance systems yielded similar results and allowed for targeted vector control applications in cases of increased virus activity. Human cases were not reported on Peloponnese in 2020.

## 1. Introduction

West Nile Virus (WNV) is a member of the *Flaviviridae* family and is maintained in nature in a bird-mosquito cycle [1]. Birds serve as reservoirs and amplifying hosts [2], since they develop sufficient levels of viremia permitting infection of ornithophilic mosquitoes during blood meals [3]. Infected mosquitoes can transmit the virus through their bites to various host species [4]. Exposed birds usually present no clinical signs, although neurological symptoms and deaths have been reported on crows and jays [5]. Horses and humans display low-level viremia [6,7,8,9] which is usually insufficient to infect mosquitoes and thus, are characterized as dead-end hosts. Humans infected with WNV can be asymptomatic, develop West Nile fever or present less frequently, severe neurologic disease [10].

The first human case was reported in Uganda in 1937 [11], while antibodies against the virus were detected in Egypt in the 1950s [12]. The virus has expanded geographically during the last three decades in countries in Africa, Europe, the Middle East, North America, and East Asia [10] and large outbreaks have occurred in Romania [13], Russia [14], the USA [15], Israel [16], and Greece [17]. Africa is believed to be the origin of WNV lineages [18], and it has been proposed that the virus spread in new areas, including Europe, through long-distance migrations of birds [19,20,21,22,23]. Establishment of enzootic WNV cycle in a region and further transmission to humans are driven by the presence of competent mosquito vectors and amplifying avian hosts, as well as the influence of environmental factors [24,25].

Human infections follow a seasonal pattern, usually from early summer to early autumn [26] and are favored by climatic conditions. Increased ambient temperatures during summer [27,28,29], high precipitation in late winter/early spring [28] and summer drought [28] play an important role in virus epidemiology. Landscape, especially existence of irrigated croplands and highly fragmented forests [28] and elevation [30] are additional risk factors for WNV outbreaks. A recent study from Greece has reported the association of high levels of precipitation during summer with the rapid dispersion of WVN in West Attica [31]. Moreover, the density of infected mosquitoes has been positively associated with the number of confirmed cases in humans [24].

In Greece, WNV infections in humans as well as deaths were initially reported on 2010 [32]. Until 2020, a total of 1360 cases including 192 fatalities were recorded from different regions [32]. Human cases were described for the first time in Peloponnese Region of Southern Greece in 2017, and the majority of them originated from Argolida Regional Unit [32]. Notably, one month before the occurrence of confirmed cases in Argolida, a reduced number of Eurasian magpies was observed along with the presence of dead wild birds. Among the dead birds, magpies with neurologic signs were identified and a WNV lineage 2 strain was isolated [33]. Two human cases were described in 2018 and none during the following years [32].

The increased number of WNV outbreaks in the last decade dictates the establishment and maintenance of surveillance systems for quantifying WNV activity levels, assessing public health risk and guiding vector control interventions. In Greece, WNV surveillance in humans is supervised by the National Public Health Organization (NPHO) and in non-human vertebrate hosts by the Ministry of Rural Development and Food. Every year, blood samples obtained from equines with clinical signs compatible to WNV infection and from sentinel horses, are tested for the presence of virus or/and virus-specific antibodies [34]. The Laboratory of Microbiology and Parasitology, Faculty of Veterinary Science, University of Thessaly and the Ministry of Rural Development have concluded a Memorandum of Cooperation for WNV surveillance in wild bird species. Specimens from susceptible dead birds as well as from the living ones of high-risk areas are examined for virus existence [34]. Many countries have implemented programs for WNV monitoring in humans, and/or vectors, and/or avian hosts and/or horses. The integration of multiple surveillance targets represents an essential step for designing effective WNV vector management strategies, though these programs can display difficulties in implementation and sustainability [35].

To reduce the potential hazard of human infections, Peloponnese Region (Southern Greece) implemented an active WNV surveillance program targeting avian hosts and mosquito vectors during 2019–2020. In response to WNV detection (in birds or mosquitoes), mosquito control intervensions were intensified prioritizing areas with increased virus circulation, while the appropriate ecological niches of WNV presence were predicted by spatial analysis. More specifically, in addition to the standard larviciding operations focusing on public areas, larviciding treatments were expanded into private properties and were integrated, as needed, with adulticiding interventions. Public awareness campaigns were also reinforced to ensure that citizens apply the appropriate personal protection measures. The current study was conducted in the context of this program, in order to provide a better insight in WNV ecology by identifying competent wild bird hosts, investigating the associations between environmental factors and WNV occurrence in wild birds and mosquito vectors, and predicting potential suitable areas for future surveillance programs.

## 2. Materials and Methods

### 2.1. Study Area

The Peloponnese Region is located in the southern land extremity of Greece (Latitude: 37°20′35.40′′ N, Longitude: 22°21′4.79′′ E). It has a hot-summer Mediterranean climate (Csa: Köppen climate classification), mainly characterized by hot, dry summers and mild, wet winters [36].

**Figure 1:**
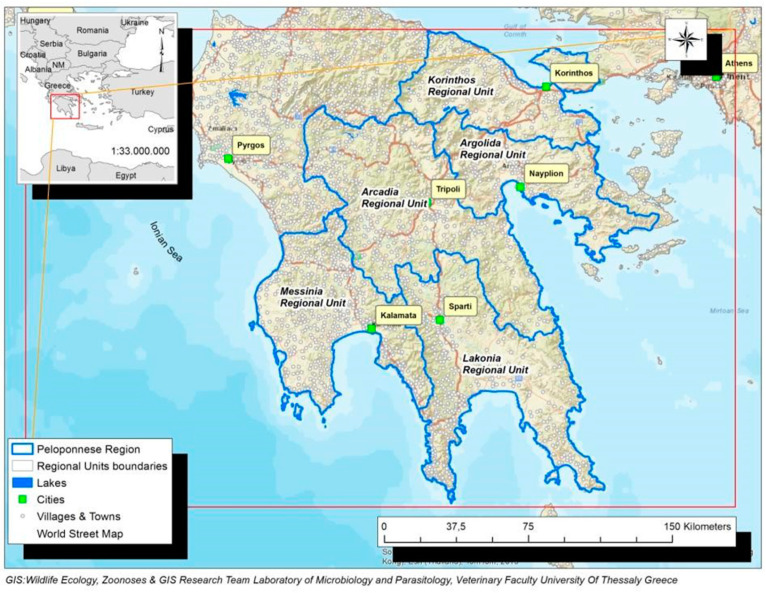
Map of Peloponnese Region in Southern Greece. The boundaries of the Regional Units of Argolida, Arcadia, Korinthia, Lakonia and Messinia are depicted with blue color.

Peloponnese Region covers a total area of 15,490 km^2^ that represents 11.7% of Greek territory and has a population of 577,903 which corresponds to 5.34% of the total national population. Soils are characterized as 50.1% mountainous, 19.9% lowlands and semi-mountainous 30%. It comprises of Regional Units of Argolida, Arcadia, Korinthia, Lakonia and Messinia which consist of 26 municipalities (Figure 1).

### 2.2. Collection of Biological Material from Wild Birds

During 2019–2020, ornithological observations were performed in Peloponnese Region to monitor and record wild bird species. Recording techniques included point-count stations, line-transects and direct count stations. Research was focused on resident species that had a relatively small home range and could act as reservoir of WNV, as well as on migratory species that could introduce new virus strains in the region.

Capturing of wild birds was performed in natural ecosystems, suburban and urban areas by using small cage traps (20 cm × 30 cm × 20 cm), Larsen Traps (60 cm × 50 cm × 50 cm), Multi Catch Larsen Trap (160 cm × 200 cm × 150 cm), groundnets and mistnets. Traps were moved periodically to different locations to increase the number of collection sites. Sampling procedure included the collection of oropharyngeal swabs (Copan). Following specimen collection, birds were released into their natural habitats according to the prerequisites of the Greek Legislation. During fieldwork, only fresh carcasses (*n* = 4 for the year 2019 and *n* = 8 for 2020) were gathered alongside the road network. Necropsies were performed and tissue samples (i.e., brain, liver, spleen, kidney) were collected and stored at −70 °C for further examination. Global Positioning System (GPS) units were used to record all sampling sites.

Biological materials were collected from 25 different species of which 15 were resident, five were migratory and five partial-migratory (Tables S1 and S2). Overall, 493 wild birds were sampled; 144 from April to November of 2019 and 349 from January to November of 2020. Specimens were retrieved from Eurasian magpies (*n* = 350), house sparrows (*n* = 59), Eurasian jays (*n* = 17), common blackbirds (*n* = 14), hooded crows (*n* = 11), great tits (*n* = 9), Eurasian tree sparrows (*n* = 4), Spanish sparrows (*n* = 3), common chiffchaffs (*n* = 3), Eurasian collared doves (*n* = 3), song thrushes (*n* = 2), jackdaws (*n* = 2), rock doves (*n* = 2), common whitethroats (*n* = 2), Eurasian eagle-owls (*n* = 2), rock partridge (*n* = 1), mallard (*n* = 1), common buzzard (*n* = 1), yellow-legged gull (*n* = 1), barn swallow (*n* = 1), purple heron (*n* = 1), tawny owl (*n* = 1), Sardinian warbler (*n* = 1), common swift (*n* = 1) and little owl (*n* = 1). Sample collection from wild bird species is described in detail in Table S1.

### 2.3. Molecular Detection of WNV in Wild Birds

Viral RNA was extracted from oropharyngeal swabs (GRS Total RNA kit—Blood and Cultured Cells, Grisp, Porto, Portugal) or 20 mg of brain tissue samples (GRS Total RNA kit—Tissue, Grisp) according to the manufacturer’s instruction. Notably, swabs were immersed in 500 μL of PCR grade water, were shaken and squeezed to the sides of the tube to extract the liquid prior to further proceeding.

Reverse transcription was performed using hexaprimers from a commercial cDNA synthesis kit (Xpert cDNA Synthesis kit, Grisp). A nested PCR targeting the NS3 region of WNV lineage 2 was used to amplify a 423 bp fragment [37]. Products of the second reaction were visualized by electrophoresis on 2% agarose gel and amplicon sizes were determined using a 100 bp DNA marker. Sanger sequencing (3730 xl DNA Analyzer, Applied Biosystems, Foster City, CA, USA) was performed on the first PCR positive samples by using the primers WN-NS3up2 and WN-NS3do2 (primers of the second PCR round) [37] to confirm the presence of WNV

### 2.4. Mosquito Sampling and Identification

An adult mosquito surveillance network with mosquito trapping devices (CDC light traps baited with CO_2_ in 35 fixed locations) was established across all regional units of Peloponnese targeting a wide-range of environments (agricultural, residential, natural) from mid-May to mid-October (2019–2020) (Table S3). All traps were operated bi-weekly and simultaneously across all fixed locations (traps were activated for approximately 15 h from 5.30 in the evening until 8.30 in the morning). Sampling locations were prioritized after consideration of the history of WNV circulation in birds and humans from the previous seasons, the presence and abundance of competent mosquito vectors/breeding sites and their proximity to residential areas and natural wetlands (aiming to target the early amplification cycle of the virus in nature). Mosquitoes were identified morphologically to species level according to Becker et al. [38].

### 2.5. Molecular Detection of WNV in Mosquitoes

Upon identification mosquitoes were divided into pools (up to 50 individuals per pool) according to species, sampling site and date. All mosquito pools were preserved at −80 °C until further analysis. For RNA extraction mosquitoes were homogenized using a piston pestle, applying the NucleoZol protocol (MACHEREY-NAGEL, Düren, Germany), following the manufacturer’s recommended instructions and diluted in a final volume of 80 μL ultrapure water. Quality and concentration of the extracted RNA were estimated in a BioSpec-nano spectrophotometer (Shimadzu Corporation, Kyoto, Japan). Examination of the presence of West Nile Virus (WNV) genomic RNA within the extracted RNA from each pool was initially performed applying a one-step multiplex Real Time reverse transcription-PCR (RT-PCR) TaqMan assay, established by Del Amo et al. [39]. Briefly, reactions were performed in 10-μL total volumes, containing approximately 50–100 ng extracted RNA, 5 μL 2× One Step PrimeScript III RT-qPCR Mix (TaKaRa Bio Inc., Shiga, Japan), 4 pmol of each one of the primer pair WN-LCV-F1—WN-LCV-R1, 1 pmol of each one of the MGB labeled probes WN-LCV-S1 and WN-LCV-S2 and RNase free water up to the final volume. The following conditions were applied for the reactions: after an initial step at 45 °C for 5 min and a subsequent denaturation step at 95 °C for 10 s, 45 cycles were performed of 10 at 95 °C for denaturation of the produced cDNA and 30 s at 60 °C for simultaneous annealing and extension. Confirmed WNV positive mosquito pools were used as positive control. Distilled water was used as negative control. The presence of WNV in positive scored pools was cross-validated and confirmed by a RT-PCR followed by a nested PCR developed and described by Shi et al. [40], with conditions and volumes of ingredients as described above.

### 2.6. Environmental Variables

Environmental variables (Table S4) consisted of climatic conditions, topography and human activities. Climate indices were derived from the WorldClim version 1.4. [41], while digital elevation model was extracted from CGIAR-CSI GeoPortal [42] and hydrological data were retrieved from HydroSHEDS [43]. Human population density and 44 categories of land uses (Table S5) were downloaded from the European Environmental Agency [44,45]. Livestock (sheep, goats, cattle) densities were retrieved from FAO [46] and normalized difference vegetation index (NDVI) was extracted from the Copernicus European earth monitoring program [47]. In this case, 14 environmental layers were created for the analysis by using ArcGIS 10·1 GIS software (ESRI, Redlands, CA, USA). Data sets were converted to a common projection map extent and resolution prior to use in the modelling program.

### 2.7. Ecological Niche Modeling (ENM) for Wild Birds and Mosquitoes

The principle of maximum entropy was applied to predict the appropriate ecological niches of WNV occurrence in wild birds and mosquitoes by using MaxEnt 3.3.3 software [48,49]. MaxEnt is a method that requires presence data, utilizes continuous and categorical data and includes efficient deterministic algorithms and mathematical definitions [48,49]. Two separate analyses were performed to associate 14 environmental variables with the locations of positive WNV wild birds and mosquito vectors, respectively.

Occurrence points were separated into training (75%) and testing (25%) data. Each predictive model was created by using training data, while its accuracy was assessed by using testing data. The performance of each model was estimated by the value in the area under the curve (AUC) of the receiver operating characteristics (ROC) curve for both training and testing data. The fitness of the model improved, as AUC value was increasing from 0.5 to 1 (AUC = 0.5; random prediction, AUC = 1; perfect performance).

Percent contribution and permutation importance were determined to assess the contributions of the environmental variables to the models. Percent contribution values were estimated heuristically. Permutation importance was calculated on the final model in order to evaluate the contribution of each variable by randomly re-ordering its values and calculating the resulting decrease in training AUC. The higher the decrease (in proportion to the rate of reduction/decrease), the model depended on the specific variable. Values were normalized to percentages [49].

Jackknife test option was applied to estimate which environmental variables were most important in the predictive models. This was performed by excluding each variable in turn and fitting the model with the remaining ones. A model was created using each variable individually. Finally, a full model was created using all variables.

Response curves were created for the variables with the higher predictive values, aiming to estimate the change in the logistic prediction as one variable varied, while all others were held at their average sample value.

Habitat suitability maps were constructed using different suitability levels ranging from unsuitable to highly suitable.

## 3. Results

### 3.1. Molecular Detection of WNV in Wild Birds

A total of 493 wild birds belonging to 25 species were molecularly screened for the presence of WNV in Peloponnese Region from 2019 until 2020. Viral RNA was not detected in the wild birds (0/144) during 2019, but it was reported on 46 birds (46/349, 13.2%) during 2020. The 46 positive birds were classified in 10 species, which were further characterized according to their migratory status, as resident (*n* = 7), partially migratory (*n* = 1) and migratory (*n* = 2). Positive specimens from resident species were obtained from all Regional Units, whereas positive samples from partial migratory and migratory species were collected only from Regional Unit of Messinia. Virus presence was demonstrated on Eurasian magpies (*n* = 30), house sparrows (*n* = 5), great tits (*n* = 3), Spanish sparrows (*n* = 2), Eurasian jays (*n* = 1), jackdaws (*n* = 1), purple herons (*n* = 1), little owls (*n* = 1), tawny owls (*n* = 1) and common whitethroats (*n* = 1). Moreover, the virus was absent from all the birds (*n* = 8) that were found dead on the road network. The data are summarized in Table 1. 

### 3.2. Molecular Detection of WNV in Pools of Mosquito Vectors

In total 678 mosquito pools were examined from 2019–2020, the majority of which belonged to the species *Culex pipiens* s.l. (*Cx. pipiens*). All 277 pools examined in 2019 were found negative for the presence of WNV. Among the 401 examined pools from 2020, 20 *Cx. pipiens* and two *Aedes albopictus* (*Ae. albopictus*) pools were tested positive for WNV (Table 2). Results were cross validated with both methodologies described above.

### 3.3. Predictive Ecological Niche Modeling (ENM) 

**Figure 2:**
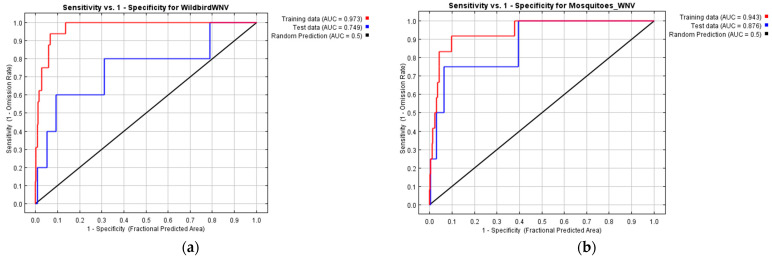
ROC curves depicting the performances of MaxEnt models (red line: training data; blue line: testing data; black line: random prediction): (**a**) Wild birds WNV model; the Area Under the Curve (AUC) had a value of 0.973 for training data and 0.749 for testing data and exceeded the value of random prediction (AUC = 0.5); (**b**) Mosquito WNV model; the Area Under the Curve (AUC) had value of 0.943 for training data and 0.876 for testing data and exceeded the value of random prediction (AUC = 0.5).

In the wild bird WNV model, positive birds (*n* = 46) were used as occurrence points and their locations were associated with 14 environmental variables to predict the appropriate ecological niches for virus presence. The model was considered to be adequately sensitive and descriptive, as ROC analysis gave an AUC value of 0.973 for training data and of 0.749 for testing data which exceeded the value 0.5 of random prediction (Figure 2a). Similarly, in the mosquito WNV model, pools of positive vectors (*n* = 22) were used as presence points. The model performed well, given that ROC analysis gave AUC values of 0.943 for training data and 0.876 for testing data that surpassed the value of random prediction (AUC = 0.5) (Figure 2b).

Altitude (58.8% percent contribution, 46.4% permutation importance) and land uses (25.7% percent contribution, 32.7% permutation importance) had a substantial importance in the wild bird WNV model, while six more variables; distance from water collections, annual mean temperature, livestock densities, May NDVI, human population density and April NDVI, gave lower contributions. Different scores were calculated for seven variables; altitude, human population density, land uses, annual mean temperature, maximum temperature of warmest month, total annual precipitation (mm), April NDVI in the mosquito WNV model, though, altitude (58.8% percent contribution, 46.4% permutation importance) gave the highest contributions. The results are presented in Table 3.

**Figure 3:**
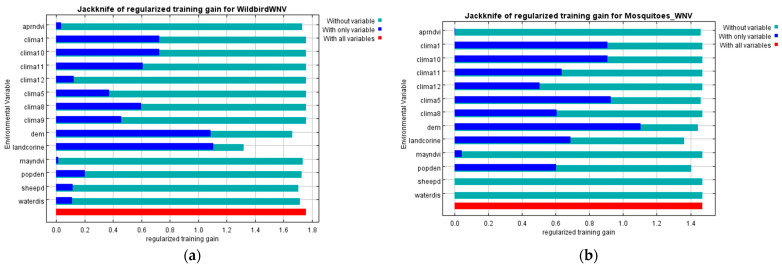
Jackknife test of the regularized training gain (light blue bar: without variable; blue bar: with only variable; red bar: with all variables): (**a**) Wild bird WNV model; (**b**) Mosquito WNV model.

Jackknife test was used to estimate the importance of each variable. In the wild bird WNV model (Figure 3a), all variables were necessary, moreover land uses (landcorine) and altitude (dem) had a relatively higher contribution. Land uses appeared to provide the most useful information and achieved the highest gain, when it was used individually, whereas gave the highest decrease in the gain when it was omitted. As for mosquito WNV model (Figure 3b), the variable of altitude (dem) gave a relatively higher contribution, having the most useful information. Furthermore, land uses (landcorine) decreased the gain the most when it was omitted and thus, contained a substantial amount of useful information that was not present in the other variables.

**Figure 4:**
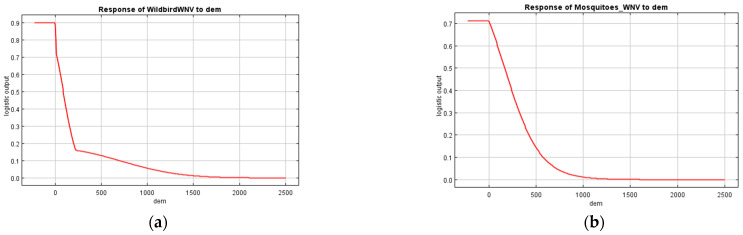
Response curves of (**a**) Wild bird WNV model and (**b**) Mosquito WNV model to altitude. Y axis: Probability of WNV presence; Maxent logistic output. X axis: Altitude (dem) indicated in meters.

**Figure 5:**
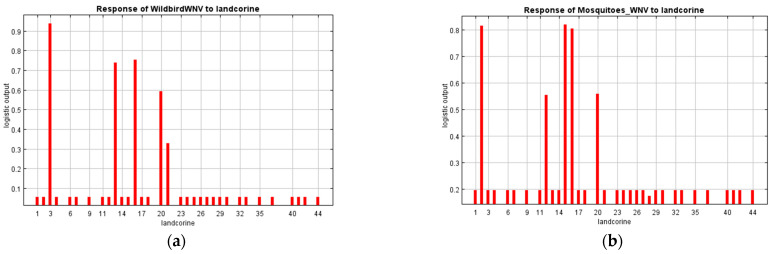
Response curves of (**a**) Wild bird WNV model and (**b**) Mosquito WNV model to land uses. Y axis: Probability of WNV presence; Maxent logistic output. X axis: 44 categories of land uses (landcorine). Red bars illustrated the positive effect of each category in model’s predictions: (**a**) Wild bird WNV model; (3) industrial or commercial units, (13) permanently irrigated land, (16) fruit trees and berry plantations, (20) complex cultivation patterns and (21) land principally occupied by agriculture; (**b**) Mosquito WNV model; (2) discontinuous urban fabric, (12) non-irrigated arable land, (15) vineyards, (16) fruit trees and berry plantations and (20) complex cultivation patterns.

Response curves were created for the variables of altitude and land uses, as they were significant predictors for both models. Suitable conditions for WNV occurrence in wild birds and mosquito vectors were identified in low altitude zones. Specifically, the probability of virus presence was higher at an elevation under 200 m (Figure 4), although positive birds and mosquitoes were recorded at 600–700 m. With respect to land uses, appropriate ecological niches for infected wild birds were identified in industrial or commercial units, permanently irrigated land, fruit trees and berry plantations, and heterogeneous agricultural areas (complex cultivation patterns and land principally occupied by agriculture, with significant areas of natural vegetation) (Figure 5). Finally, discontinuous urban fabric, non-irrigated arable land, permanent crops (vineyards, fruit trees and berry plantations), and complex cultivation patterns were recognized as suitable habitats for positive vectors (Figure 5).

**Figure 6:**
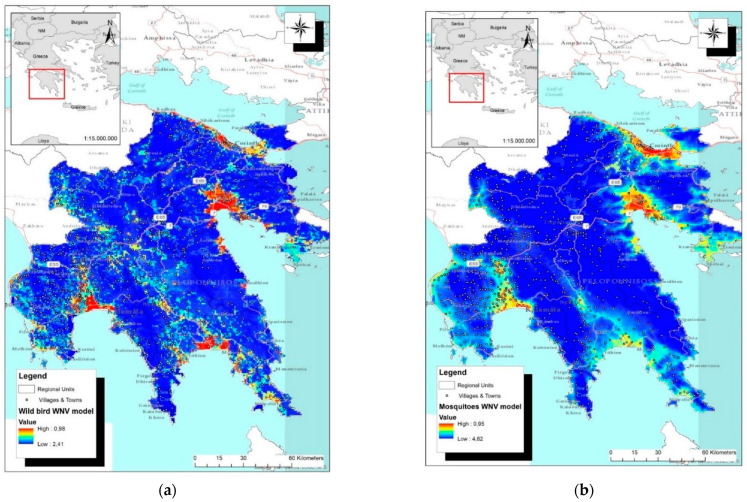
Habitat suitability maps of WNV in Peloponnese Region: The predicted probability of WNV presence was depicted with different colors (red color: highly suitable habitat; blue color: unsuitable habitat): (**a**) Wild bird WNV model; (**b**) Mosquito WNV model.

The potential geographic distribution of WNV in wild birds and mosquito vectors in Peloponnese Region was illustrated in maps (Figure 6). Highly suitable areas for virus occurrence were recognized in the coastal zone of Korinthos, the plain of Argos, the municipality of Evrotas and in western Messinia.

## 4. Discussion

The present study was conducted in the context of an active WNV surveillance program in Peloponnese Region of Southern Greece during 2019–2020. The focus was the detection of WNV in wild birds and mosquito vectors, as well as the identification of environmental factors that could favor the virus circulation and dispersion. To this end, 493 wild birds and 678 pools of mosquito vectors were molecularly screened for the presence of WNV, and even though the virus was not identified in specimens collected in 2019, it was detected in 46 of 349 wild birds and in 22 of 401 mosquito pools during 2020.

The two-year monitoring period of wild birds in Peloponnese Region demonstrated the inhabitation of resident, partial-migratory and migratory species in different ecological niches. During field work, massive deaths or presence of birds displaying neurological signs, especially of magpies, were not described in contrast to the previously reported findings from the Regional Unit of Argolida in 2017 [33].

Wild bird species display different susceptibility range to WNV infection, while members of the *Corvidae* family have been described to be more vulnerable [50,51]. Eurasian magpies were selected as the target group of sampling, considering their susceptibility [33,52,53], their ability to live in proximity to humans in a variety of habitats and their relative abundance in the area, as it was evidenced by the monitoring of wild birds. In particular, during 2020, samples from 227 magpies were collected over the entire area of Peloponnese Region and virus was detected in 30 of them (30/227 [13.22%]). Notably, the first proof of WNV circulation during 2020 was detected via a magpie sample, further supporting that this species constitutes a good early warning indicator of WNV circulation.

House sparrow is a resident species, closely associated with human habitation [54], and widely distributed in Peloponnese region. Reviewing literature demonstrated persistence of an early lineage 1 WNV strain (NY99) on house sparrows [55,56], although resistance was developed over time and increased susceptibility was reported for new circulating strains [57]. In view of the previous data, we aimed to determine whether house sparrows could be playing an important role as amplifier hosts and could be included in the list of target species for future surveillance programs. To that direction, specimens from 57 house sparrows were screened and virus presence was confirmed in five of them (5/57 [8.77%]). Identification of WNV in house sparrows indicated that this species could be included along with magpies, as a marker of virus circulation in future monitoring programs.

The risk of WNV circulation was also investigated in resident species other than magpies and house sparrows. Virus was identified in a jackdaw (1/2) and a Eurasian jay (1/12), both members of the crow family that display susceptibility to WNV infection [50,51] and inhabit the woodlands. Despite the vulnerability of jackdaws and jays, the purpose of this study was to attain an early warning of WNV circulation in order to protect public health. Therefore, our sampling effort was focused on species that share common habitats with mosquitoes and humans. Regarding the four owls tested, WNV presence was confirmed in a tawny owl and a little owl, but not in the two Eurasian eagle-owls, and to our knowledge, this finding is reported for the first time in Greece. Our results are in agreement with previous studies that refer to affected owls in North America [58], Germany and Southern Europe [59]. Furthermore, virus RNA was detected in three great tits (3/8). Based on the aforementioned data, we conclude that the detection of WNV in seven resident species supports strongly the idea of virus endemic circulation in Peloponnese region.

Assuming that migratory birds could play a significant role in the introduction of WNV in a region [19,20,21,22,23], two migratory and one partial-migratory species were tested in 2020. Unfortunately, the sample size of the screened birds was small (*n* = 10), since specimens were obtained during routine investigation of sick and injured birds, only from regional unit of Messinia which has important wetlands [60] and is located within a major migration route. Specifically, WNV exposure was confirmed in a purple heron (1/1) and a common whitethroat (1/2) which can travel long distances for wintering, while Greece is a territory of passage and/or native breeding from April to October [61,62]. In addition, two Spanish sparrows (2/3), partial-migrants that are known to visit the area of Messinia during the non-breeding season (winter) [63], yielded positive results for WNV. Nevertheless, virus occurrence in the aforesaid species provided evidence of a potential new virus import and raised concerns about its origin and dispersion.

Identification of WNV was performed in twenty pools of *Cx. pipiens* as well as in two pools of *Ae. albopictus*. The vector competence of *Cx. pipiens* is well documented in Europe, as it is considered to be the principal WNV virus vector [64]. The potential of *Ae. albopictus* mosquitoes to transmit WNV has been well established under laboratory conditions [65], however, their contribution to virus circulation under field conditions is considered limited due to their low propensity to feed on avian hosts. In respect of our findings, the contribution of *Ae. albopictus* in the ecology of WNV in Peloponnese Region remains to be clarified. Future studies should focus on the ecology of *Ae. albopictus* in natural ecosystems with well-established WNV transmission cycles in order to improve our knowledge on the feeding preferences of this species relating to avian wildlife.

The results produced via bird- and mosquito-based surveillance were in concordance, given that positive birds and vectors were not reported on 2019, though during 2020, virus presence was confirmed with both systems in the same sampling areas. When two different, parallel systems of molecular xeno-monitoring, analyzed independently by different groups of experts agree, implementation of interventions is necessary for the protection public health. In fact, the public health authorities of Peloponnese Region proceeded in campaigns for strengthening public awareness and in immediate targeted vector control operations prioritizing areas with increased virus activity. Intensification of vector control operations was sustained until no indication of WNV circulation was reported from the surveillance system. During 2020, no human cases occurred in Peloponnese, even though 145 confirmed cases including 23 deaths were reported for the rest of the country [32]. Thus, we hypothesize that the immediate and intensive vector control strategy applied by the Region of Peloponnese contributed to limiting WNV circulation below the levels of epizootic transmission. Studies are needed to investigate and quantify the impact of vector control practices on WNV circulation levels and disease transmission to humans, while bird/mosquito—based surveillance systems, as the one described here, can be used as surrogates for human cases.

It is well documented that environmental conditions can drive WNV infections, given that both avian hosts and vectors are implicated in the virus transmission cycle. Spatial analysis has been used in the past to detect the appropriate ecological niches for virus presence [66], and thus, in our study, two different MaxEnt models were applied; one for wild birds and another for mosquito vectors. Altitude and land uses were identified as significant predictors by both models. In particular, the probability of WNV detection in birds and mosquitoes was higher in the low altitude zone under 200 m, a fact which further confirms our previous results [66]. Certain categories of land uses were positively associated with the potential virus occurrence in wild birds, and especially industrial or commercial units, arable land, permanent crops and heterogeneous agricultural areas. Considering that the current surveillance program was based primarily on resident species that live closely to humans, all the above habitats provide suitable conditions for their survival by covering their basic needs for food, water and shelter. Notably, permanent crops are a natural environment with increased food availability and nesting sites for many species including corvids.

With respect to mosquito vectors, highly suitable habitats were identified in the classes of discontinuous urban fabric, arable land, permanent crops and heterogeneous agricultural areas. These niches met the criteria for mosquitoes to complete the different stages of their life cycle. To begin, a characteristic of these habitats is the existence of stagnant water (i.e., puddles, streams, cesspits), which is a prerequisite for the eggs to become adults. Furthermore, in these regions, female adults can feed on blood from humans and animals, mainly wild birds, to produce their eggs. Gonzalez et al. [67], reported that *Cx. pipiens* were more abundant in urban, peri-urban and rural areas and exhibited avian feeding preferences with great tit, common blackbird, Eurasian magpie and house sparrow being the most dominant species. Our findings are in line with the former study, as our analyses demonstrated higher probability of positive WNV wild birds and vectors in the abovementioned natural environments. In fact, we detected WNV RNA in specimens retrieved from three of the preceding species (great tit, Eurasian magpie, house sparrow) as well as from *Cx. pipiens* living in similar types of habitats.

## 5. Conclusions

West Nile Virus ecology is a complex phenomenon considering the knowledge gaps on the environmental parameters conducive to virus amplification and disease transmission. Given the little capacity to predict outbreaks, real-time bird- and mosquito-based surveillance networks, can be very useful in gauging virus circulation levels and supporting vector control decisions. A two-year period integrated bird-mosquito WNV surveillance system was implemented in the Region of Peloponnese. During 2019, virus detection as well as human cases were not reported, whereas in 2020, the surveillance system provided timely-accurate and dynamic information on virus circulation in birds and mosquitoes, further leading to the implementation of targeted vector control measures. Moreover, collected data provided a better insight on the role of bird species in virus circulation, and by processing bird- and mosquito related information, a WNV habitat suitability map was created. The identification of capable avian hosts and mosquito vectors together with the appropriate ecological niches of virus presence can be useful tools for future surveillance programs, especially in this area. To predict and mitigate the risk of disease transmission, long-term surveillance studies, as this one, can enhance gradually our understanding on the ecology of WNV.

## Figures and Tables

**Table 1:** Positive WNV wild bird species from Peloponnese Region during 2020.

No	Wild Bird Species		Migratory Status	Regional Units					Total
	Scientific Name	Common Name		Argolida	Arcadia	Korinthia	Lakonia	Messinia	
1	*Ardea purpurea*	Purple heron	Migratory	- ^1^	-	-	-	1/1 ^2^	1/1
2	*Athene noctua*	Little owl	Resident	-	-	-	-	1/1	1/1
3	*Corvus monedula*	Jackdaw	Resident	-	1/2	-	-	-	1/2
4	*Curruca communis*	Common whitethroat	Migratory	-	0/1	-	-	1/1	1/2
5	*Garrulus glandarius*	Eurasian jay	Resident	-	1/6	0/1	0/4	0/1	1/12
6	*Parus major*	Great tit	Resident	-	0/3	1/1	-	2/4	3/8
7	*Passer domesticus*	House sparrow	Resident	-	0/4	2/26 (25 + 1 ^D^)	3/16	0/11	5/57 (8.77%)
8	*Passer hispaniolensis*	Spanish sparrow	Partial migratory	-	-	-	-	2/3	2/3
9	*Pica pica*	Eurasian magpie	Resident	12/53 (51 + 2 ^D^)	3/39 (38 + 1 ^D^)	12/71 (67 + 4 ^D^)	2/36	1/28	30/227 (13.22%)
10	*Strix aluco*	Tawny owl	Resident	-	-	-	-	1/1	1/1

^1^ Sample not available. ^2^ Positive WNV birds versus tested birds. ^D^ Wild birds found dead.

**Table 2:** WNV positive mosquito pools from Peloponnese Region during 2020.

Regional Unit	Species	Number of Positive Pools/ Total Pools Examined
Argolida	*Culex pipiens*	6/137
	*Aedes albopictus*	1/12
Arcadia	*Culex pipiens*	4/75
	*Aedes albopictus*	1/10
Korinthia	*Culex pipiens*	4/27
	*Aedes albopictus*	0/3
Laconia	*Culex pipiens*	4/60
	*Aedes albopictus*	0/7
Messinia	*Culex pipiens*	2/64
	*Aedes albopictus*	0/6
Total	*Culex pipiens*	20/363
	*Aedes albopictus*	2/38

**Table 3:** Percent contribution and permutation importance of environmental variables to wild bird and mosquito WNV models.

**Wild Bird WNV Model**			
**Environmental Variable**	**Code**	**Percent Contribution (%)**	**Permutation Importance (%)**
Altitude	dem	58.8	46.4
Land uses (44 categories)	landcorine	25.7	32.7
Distance from water collections	waterdis	3.9	1.8
Annual mean temperature	clima1	3.8	2.0
Livestock densities	sheepd	3.7	3.6
May NDVI ^1^	mayndvi	2.2	3.3
Human population density	popden	1.0	7.3
April NDVI ^1^	aprndvi	0.8	2.4
**Mosquito WNV model**			
**Environmental variable**	**Code**	**Percent contribution (%)**	**Permutation importance (%)**
Altitude	dem	31.2	98.5
Human population density	popden	22.9	0
Land uses (44 categories)	landcorine	22.9	0.9
Annual mean temperature	clima1	15.7	0
Maximum temperature of warmest month	clima5	3.8	0.7
Total annual precipitation (mm)	clima12	12.3	0
April NDVI ^1^	aprndvi	1.2	0

^1^ NDVI: normalized difference vegetation index.

## Data Availability

Data of this study are presented within the manuscript and the Supplementary Materials.

## Supplementary Materials

The following supporting information can be downloaded at: https://www.mdpi.com/article/10.3390/microorganisms10071328/s1, Table S1: Sample collection from wild bird species in Peloponnese Region during 2019–2020; Table S2: Collection sites of wild birds; Table S3: Collection sites of mosquito pools; Table S4: Environmental variables; Table S5: Categories of land uses.
