# Supplementary material for: West Nile Virus Occurrence and Ecological Niche Modeling in Wild Bird Species and Mosquito Vectors: An Active Surveillance Program in the Peloponnese Region of Greece
Source: Microorganisms. 2022 Jun 30;10(7):1328. doi: 10.3390/microorganisms10071328 (PMC9320058; doi:10.3390/microorganisms10071328)
Supplement: Supplementary file 1 [file microorganisms-10-01328-s001.zip › microorganisms-1760224-supplementary.pdf]

Table S1. Sample collection from wild bird species in Peloponnese Region during 2019–2020.

Wild Bird Species			Migratory status	Regional Units of Peloponnese Region					
No	Scientific name	Common name		Total	Argolida	Arcadia	Korinthia	Lakonia	Messinia
1	*Alectoris graeca*	Rock partridge	Resident	1	-	-	1	-	-
2	*Anas platyrhynchos*	Mallard	Partial migratory	1	1	-	-	-	-
3	*Apus apus*	Common swift	Migratory	1	-	-	-	-	1
4	*Ardea purpurea*	Purple heron	Migratory	1	-	-	-	-	1
5	*Athene noctua*	Little owl	Resident	1	-	-	-	-	1
6	*Bubo bubo*	Eurasian eagle-owl	Resident	2	-	-	-	-	2
7	*Buteo buteo*	Common buzzard	Partial migratory	1	-	-	-	-	1
8	*Columba livia*	Rock dove	Resident	2	-	-	-	-	2
9	*Corvus cornix*	Hooded crow	Resident	11	1	3	1	3	3
10	*Corvus monedula*	Jackdaw	Resident	2		2		-	-
11	*Curruca communis*	Common whitethroat	Migratory	2	-	1	-	-	1
12	*Curruca melanocephala*	Sardinian warbler	Resident	1	-	-	-	-	1
13	*Garrulus glandarius*	Eurasian jay	Resident	17	3	6	3	4	1
14	*Hirundo rustica*	Barn swallow	Migratory	1	-	-	-	-	1
15	*Larus michahellis*	Yellow-legged gull	Resident	1	-	-	1	-	-
16	*Parus major*	Great tit	Resident	9	1	3	1	-	4
17	*Passer domesticus*	House sparrow	Resident	59 (58+1[D])	-	5	26 (25+1[D])	16	12
18	*Passer hispaniolensis*	Spanish sparrow	Partial migratory	3	-	-	-	-	3
19	*Passer montanus*	Eurasian tree sparrow	Resident	4	-	-	-	-	4
20	*Phylloscopus collybita*	Common chiffchaff	Partial migratory	3	-	2	-	-	1
21	*Pica pica*	Eurasian magpie	Resident	350 (339+11[D])	71 (70+3[D])	67 (65+2[D])	109 (104+6[D])	50	53
22	*Streptopelia decaocto*	Eurasian collared dove	Resident	3	-	-	3	-	-
23	*Strix aluco*	Tawny owl	Resident	1	-	-	-	-	1
24	*Turdus merula*	Common blackbird	Partial migratory	14	1	1	2	7	3
25	*Turdus philomelos*	Song thrush	Migratory	2	1	-	-	1	-

[D] Wild birds found dead

Table S2. Collection sites of wild birds

Bird Code	Year	Regional Unit	Region	Species	PCR result	WGS84_X	WGS84_Y
WB1	2019	Argolida	Inachos	*Parus major*	0	22,82394	37,66967
WB2	2019	Argolida	Argos	*Pica pica*	0	22,74262	37,63296
WB3	2019	Argolida	Monastiraki	*Pica pica*	0	22,75054	37,69997
WB4	2019	Argolida	Koutsopodi	*Pica pica*	0	22,72178	37,68528
WB5	2019	Argolida	Neo Iraio	*Pica pica*	0	22,76800	37,66681
WB6	2019	Argolida	Argos	*Pica pica*	0	22,70256	37,64525
WB7	2019	Argolida	Limnes	*Pica pica*	0	22,96747	37,77093
WB8	2019	Argolida	Nea Kios	*Pica pica*	0	22,74700	37,59200
WB9	2019	Argolida	Karya	*Pica pica*	0	22,62527	37,62620
WB10	2019	Argolida	Inachos	*Pica pica*	0	22,75500	37,66600
WB11	2019	Argolida	Tirintha	*Pica pica*	0	22,78500	37,60000
WB12	2019	Argolida	Skafidaki	*Pica pica*	0	22,71456	37,56978
WB13	2019	Argolida	Koutsopodi	*Pica pica*	0	22,87303	37,70910
WB14	2019	Argolida	Fixti	*Pica pica*	0	22,71944	37,72831
WB15	2019	Argolida	Kranidi	*Pica pica*	0	23,14200	37,38700
WB16	2019	Argolida	Dimaina	*Pica pica*	0	23,06700	37,69900
WB17	2019	Argolida	Laloukas	*Pica pica*	0	22,77800	37,62600
WB18	2019	Argolida	Asini	*Pica pica*	0	22,86805	37,55083
WB19	2019	Argolida	Skafidaki	*Pica pica*	0	22,67800	37,56400
WB20	2019	Argolida	Tirintha	*Garrulus glandarius*	0	22,83500	37,61100
WB21	2019	Argolida	Laloukas	*Garrulus glandarius*	0	22,79200	37,62900
WB22	2019	Argolida	Laloukas	*Garrulus glandarius*	0	22,79200	37,61500
WB23	2019	Argolida	Laloukas	*Corvus cornix*	0	22,77875	37,63152
WB24	2019	Argolida	Tirintha	*Anas platyrhynchos*	0	22,80400	37,60300
WB25	2019	Arcadia	Merkovouni	*Pica pica*	0	22,37789	37,54395
WB26	2019	Arcadia	Makri	*Pica pica*	0	22,32539	37,47702
WB27	2019	Arcadia	Asea	*Pica pica*	0	22,28578	37,40671
WB28	2019	Arcadia	Thana	*Pica pica*	0	22,34800	37,47600
WB29	2019	Arcadia	Ampelakia	*Pica pica*	0	22,23319	37,39314
WB30	2019	Arcadia	Nestani	*Pica pica*	0	22,84415	37,61308
WB31	2019	Arcadia	Makri	*Pica pica*	0	22,46416	37,47844
WB32	2019	Arcadia	Palladio	*Pica pica*	0	22,35646	37,46296
WB33	2019	Arcadia	Makri	*Pica pica*	0	22,33595	37,46486
WB34	2019	Arcadia	Ampelakia	*Pica pica*	0	22,45391	37,60655
WB35	2019	Arcadia	Arachamites	*Pica pica*	0	22,31644	37,46778
WB36	2019	Arcadia	Asea	*Pica pica*	0	22,24482	37,44717
WB37	2019	Arcadia	Ampelakia	*Pica pica*	0	22,29042	37,38400
WB38	2019	Arcadia	Thana	*Pica pica*	0	22,36200	37,47600
WB39	2019	Arcadia	Thana	*Pica pica*	0	22,37300	37,46300
WB40	2019	Arcadia	Manari	*Pica pica*	0	22,32500	37,40300
WB41	2019	Arcadia	Nestani	*Pica pica*	0	22,40400	37,59700
WB42	2019	Arcadia	Thana	*Pica pica*	0	22,34814	37,47601
WB43	2019	Arcadia	Tripoli	*Pica pica*	0	22,39700	37,51000
WB44	2019	Arcadia	Astros	*Pica pica*	0	22,75121	37,40374
WB45	2019	Arcadia	Agiogritika	*Pica pica*	0	22,47000	37,49200
WB46	2019	Arcadia	Silimni	*Pica pica*	0	22,29900	37,51600
WB47	2019	Arcadia	Thana	*Pica pica*	0	22,39700	37,51000
WB48	2019	Arcadia	Tripoli	*Pica pica*	0	22,35300	37,50400
WB49	2019	Arcadia	Tripoli	*Pica pica*	0	22,36877	37,49269
WB50	2019	Arcadia	Merkovouni	*Pica pica*	0	22,37467	37,54819
WB51	2019	Arcadia	Papari	*Pica pica*	0	22,25954	37,37393

WB52	2019	Arcadia	Xoremi	*Pica pica*	0	22,07213	37,38248
WB53	2019	Arcadia	Chrisovitsi	*Corvus cornix*	0	22,23200	37,51000
WB54	2019	Arcadia	Palladio	*Corvus cornix*	0	22,35339	37,46354
WB55	2019	Arcadia	Asea	*Passer domesticus*	0	22,28021	37,40335
WB56	2019	Korinthia	Athikia	*Pica pica*	0	22,98923	37,81015
WB57	2019	Korinthia	Galataki	*Pica pica*	0	22,92916	37,81290
WB58	2019	Korinthia	Examilia	*Pica pica*	0	22,95528	37,91738
WB59	2019	Korinthia	Assos	*Pica pica*	0	22,83100	37,92600
WB60	2019	Korinthia	Examilia	*Pica pica*	0	22,91100	37,87900
WB61	2019	Korinthia	Galataki	*Pica pica*	0	22,97500	37,85300
WB62	2019	Korinthia	Solomos	*Pica pica*	0	22,89705	37,88008
WB63	2019	Korinthia	Isthmia	*Streptopelia decaocto*	0	23,00517	37,90434
WB64	2019	Korinthia	Athikia	*Pica pica*	0	22,97966	37,86447
WB65	2019	Korinthia	Galataki	*Pica pica*	0	22,98923	37,86653
WB66	2019	Korinthia	Ancient Korinthos	*Pica pica*	0	22,94831	37,91257
WB67	2019	Korinthia	Loutraki-Petrachora	*Pica pica*	0	23,01525	37,96149
WB68	2019	Korinthia	Xilokastro	*Pica pica*	0	22,62880	38,07042
WB69	2019	Korinthia	Galataki	*Pica pica*	0	22,77678	37,86997
WB70	2019	Korinthia	Xilokeriza	*Pica pica*	0	22,94300	37,87700
WB71	2019	Korinthia	Xilokeriza	*Pica pica*	0	22,94900	37,87700
WB72	2019	Korinthia	Sikiona	*Pica pica*	0	22,71600	37,98400
WB73	2019	Korinthia	Examilia	*Pica pica*	0	22,95533	37,91419
WB74	2019	Korinthia	Korinthos	*Pica pica*	0	22,83325	37,92946
WB75	2019	Korinthia	Examilia	*Pica pica*	0	22,94691	37,91250
WB76	2019	Korinthia	Korinthos	*Pica pica*	0	22,94893	37,92685
WB77	2019	Korinthia	Examilia	*Pica pica*	0	22,94888	37,91258
WB78	2019	Korinthia	Velos	*Pica pica*	0	22,75700	37,98278
WB79	2019	Korinthia	Zeugolatio	*Pica pica*	0	22,79565	37,92201
WB80	2019	Korinthia	Xilokeriza	*Pica pica*	0	22,87082	37,90433
WB81	2019	Korinthia	Examilia	*Pica pica*	0	22,95062	37,91188
WB82	2019	Korinthia	Galataki	*Pica pica*	0	22,97600	37,84900
WB83	2019	Korinthia	Xilokeriza	*Pica pica*	0	22,99137	37,86143
WB84	2019	Korinthia	Examilia	*Pica pica*	0	22,94678	37,91308
WB85	2019	Korinthia	Examilia	*Pica pica*	0	22,95062	37,91188
WB86	2019	Korinthia	Examilia	*Pica pica*	0	22,94900	37,91300
WB87	2019	Korinthia	Korinthos	*Pica pica*	0	22,94261	37,92566
WB88	2019	Korinthia	Korinthos	*Pica pica*	0	22,94510	37,92356
WB89	2019	Korinthia	Xilokeriza	*Pica pica*	0	22,94734	37,88049
WB90	2019	Korinthia	Galataki	*Pica pica*	0	22,99451	37,84710
WB91	2019	Korinthia	Kefalari	*Pica pica*	0	22,53400	37,91900
WB92	2019	Korinthia	Sikiona	*Pica pica*	0	22,72807	37,98475
WB93	2019	Korinthia	Assos	*Pica pica*	0	22,81503	37,93655
WB94	2019	Korinthia	Xilokeriza	*Pica pica*	0	22,94734	37,88049
WB95	2019	Korinthia	Ancient Nemea	*Garrulus glandarius*	0	22,72194	37,80857
WB96	2019	Korinthia	Chiliomodi	*Garrulus glandarius*	0	22,81007	37,82497
WB97	2019	Korinthia	Zeugolatio	*Turdus merula*	0	22,94918	37,87547
WB98	2019	Korinthia	Goura	*Corvus cornix*	0	22,33400	37,92500
WB99	2019	Korinthia	Sarantapicho	*Alectoris chukar*	0	22,36553	38,03218
WB100	2019	Lakonia	Klada	*Pica pica*	0	22,41968	37,10822
WB101	2019	Lakonia	Sparti	*Pica pica*	0	22,42390	37,09762
WB102	2019	Lakonia	Neapoli	*Pica pica*	0	23,06700	36,50700

WB103	2019	Lakonia	Geraki	*Pica pica*	0	22,70200	36,98300
WB104	2019	Lakonia	Agios Dimitrios Zarakos	*Pica pica*	0	22,81500	36,94100
WB105	2019	Lakonia	Asteri	*Pica pica*	0	22,75200	36,80900
WB106	2019	Lakonia	Skala	*Pica pica*	0	22,66200	36,86000
WB107	2019	Lakonia	Sparti	*Pica pica*	0	22,42700	37,03300
WB108	2019	Lakonia	Krokees	*Pica pica*	0	22,55300	36,88900
WB109	2019	Lakonia	Peristeri	*Pica pica*	0	22,66600	36,87900
WB110	2019	Lakonia	Papadianika	*Pica pica*	0	22,86500	36,72900
WB111	2019	Lakonia	Vlachioti	*Pica pica*	0	22,71900	36,85800
WB112	2019	Lakonia	Peristeri	*Pica pica*	0	22,67516	36,85939
WB113	2019	Lakonia	Asteri	*Pica pica*	0	22,73472	36,82668
WB114	2019	Lakonia	Asteri	*Turdus merula*	0	22,74700	36,81000
WB115	2019	Lakonia	Platanos	*Corvus cornix*	0	22,48700	36,77700
WB116	2019	Lakonia	Elaia	*Corvus cornix*	0	22,81700	36,74900
WB117	2019	Messinia	Messini	*Pica pica*	0	21,99898	37,05576
WB118	2019	Messinia	Verga	*Pica pica*	0	22,16140	37,00989
WB119	2019	Messinia	Loggas	*Pica pica*	0	21,91037	36,86449
WB120	2019	Messinia	Verga	*Pica pica*	0	22,15700	37,01200
WB121	2019	Messinia	Valira	*Pica pica*	0	21,98400	37,15700
WB122	2019	Messinia	Verga	*Pica pica*	0	22,16500	36,98700
WB123	2019	Messinia	Messini	*Pica pica*	0	21,98200	37,04200
WB124	2019	Messinia	Messini	*Pica pica*	0	21,97600	37,05200
WB125	2019	Messinia	Karteroli	*Pica pica*	0	21,98500	37,07900
WB126	2019	Messinia	Xirokampos	*Pica pica*	0	21,68400	37,22400
WB127	2019	Messinia	Kopanaki	*Pica pica*	0	21,81200	37,28100
WB128	2019	Messinia	Dorio	*Pica pica*	0	21,84900	37,29600
WB129	2019	Messinia	Valira	*Pica pica*	0	21,98300	37,17700
WB130	2019	Messinia	Platea	*Pica pica*	0	22,01600	37,15900
WB131	2019	Messinia	Lampaini	*Pica pica*	0	21,96800	37,15400
WB132	2019	Messinia	Mauromati Pamisou	*Pica pica*	0	21,99100	37,06800
WB133	2019	Messinia	Messini	*Pica pica*	0	21,97872	37,05209
WB134	2019	Messinia	Loggas	*Pica pica*	0	21,91400	36,86000
WB135	2019	Messinia	Kompoi	*Pica pica*	0	21,92200	36,82200
WB136	2019	Messinia	Evaggelismos	*Pica pica*	0	21,77700	36,81700
WB137	2019	Messinia	Psariou	*Pica pica*	0	21,87500	37,32200
WB138	2019	Messinia	Karteroli	*Pica pica*	0	21,98381	37,08021
WB139	2019	Messinia	Loggas	*Pica pica*	0	21,89941	36,87285
WB140	2019	Messinia	Kyparissia	*Pica pica*	0	21,66012	37,23522
WB141	2019	Messinia	Mauromati Pamisou	*Pica pica*	0	21,99138	37,06250
WB142	2019	Messinia	Arfara	*Corvus cornix*	0	22,50709	37,17230
WB143	2019	Messinia	Arfara	*Corvus cornix*	0	22,04288	37,15022
WB144	2019	Messinia	Asprochoma	*Passer domesticus*	0	22,07600	37,04700
WB145	2020	Argolida	Mili	*Pica pica*	0	22,95823	37,55128
WB146	2020	Argolida	Iria	*Pica pica*	0	22,69547	37,53325
WB147	2020	Argolida	Drepano	*Pica pica*	0	22,40149	37,59628
WB148	2020	Argolida	Nea Tirintha	*Pica pica*	0	22,63983	37,61765
WB149	2020	Argolida	Fregeni	*Pica pica*	0	22,64633	37,62223
WB150	2020	Argolida	Karia	*Pica pica*	0	22,57521	37,64342
WB151	2020	Argolida	Dalamanara	*Pica pica*	0	22,64200	37,62900

WB152	2020	Argolida	Anifi	*Pica pica*	0	22,74741	37,66682
WB153	2020	Argolida	Mideas	*Pica pica*	0	22,69831	37,70110
WB154	2020	Argolida	Koutsopodi	*Pica pica*	0	22,84294	37,66681
WB155	2020	Argolida	Kiveri	*Turdus merula*	0	22,80818	37,64907
WB156	2020	Argolida	Limnes	*Turdus philomelos*	0	22,81152	37,64532
WB157	2020	Argolida	Malandreni	*Pica pica*	0	22,81003	37,64718
WB158	2020	Argolida	Karia	*Pica pica*	0	22,64200	37,62900
WB159	2020	Argolida	Mili	*Pica pica*	0	22,69547	37,53325
WB160	2020	Argolida	Mideas	*Pica pica*	0	22,69831	37,70110
WB161	2020	Argolida	Koutsopodi	*Pica pica*	0	22,70900	37,70200
WB162	2020	Argolida	Argos	*Pica pica*	0	22,74262	37,63296
WB163	2020	Argolida	Nea Tirintha	*Pica pica*	0	22,81655	37,61094
WB164	2020	Argolida	Argos	*Pica pica*	0	22,72344	37,64966
WB165	2020	Argolida	Karia	*Pica pica*	0	22,77845	37,62623
WB166	2020	Argolida	Epidavros	*Pica pica*	0	23,12927	37,62606
WB167	2020	Argolida	Dalamanara	*Pica pica*	1	22,78048	37,61466
WB168	2020	Argolida	Dalamanara	*Pica pica*	0	22,77904	37,61506
WB169	2020	Argolida	Poulakida	*Pica pica*	1	22,77048	37,61933
WB170	2020	Argolida	Dalamanara	*Pica pica*	0	22,77048	37,61933
WB171	2020	Argolida	Mideas	*Pica pica*	0	22,84294	37,66681
WB172	2020	Argolida	Mideas	*Pica pica*	0	22,80818	37,64907
WB173	2020	Argolida	Mideas	*Pica pica*	0	22,80818	37,64907
WB174	2020	Argolida	Dalamanara	*Pica pica*	1	22,78052	37,61446
WB175	2020	Argolida	Dalamanara	*Pica pica*	1	22,78068	37,61422
WB176	2020	Argolida	Dalamanara	*Pica pica*	1	22,76308	37,61958
WB177	2020	Argolida	Dalamanara	*Pica pica*	1	22,76313	37,61957
WB178	2020	Argolida	Dalamanara	*Pica pica*	1	22,76394	37,61933
WB179	2020	Argolida	Dalamanara	*Pica pica*	1	22,76214	37,61927
WB180	2020	Argolida	Poulakida	*Pica pica*	1	22,80747	37,64834
WB181	2020	Argolida	Poulakida	*Pica pica*	1	22,80747	37,64834
WB182	2020	Argolida	Poulakida	*Pica pica*	1	22,80863	37,64532
WB183	2020	Argolida	Poulakida	*Pica pica*	1	22,80863	37,64532
WB184	2020	Argolida	Fichti	*Pica pica*	0	22,73045	37,72247
WB185	2020	Argolida	Fichti	*Pica pica*	0	22,73000	37,75500
WB186	2020	Argolida	Fichti	*Pica pica*	0	22,71920	37,72951
WB187	2020	Argolida	Drepanos	*Pica pica*	0	22,89197	37,53564
WB188	2020	Argolida	Kefalari	*Pica pica*	0	22,67633	37,59008
WB189	2020	Argolida	Dalamanara	*Pica pica*	0	22,76587	37,62585
WB190	2020	Argolida	Dalamanara	*Pica pica*	0	22,77357	37,61337
WB191	2020	Argolida	Anifi	*Pica pica*	0	22,78478	37,65931
WB192	2020	Argolida	Koutsopodi	*Pica pica*	0	22,69485	37,68709
WB193	2020	Argolida	Argos	*Pica pica*	0	22,74598	37,62732
WB194	2020	Argolida	Laloukas	*Pica pica*	0	22,77805	37,63255
WB195	2020	Argolida	Mideas	*Pica pica*	0	22,80818	37,64907
WB196	2020	Argolida	Mideas	*Pica pica*	0	22,80818	37,64907
WB197	2020	Argolida	Koutsopodi	*Pica pica*	0	22,72261	37,69029
WB198	2020	Argolida	Koutsopodi	*Pica pica*	0	22,71823	37,66945
WB199	2020	Argolida	Inachos	*Pica pica*	0	22,82394	37,66967
WB200	2020	Arcadia	Asea	*Corvus corone cornix*	0	22,28311	37,40891
WB201	2020	Arcadia	Vlachokerasia	*Pica pica*	0	22,30570	37,36610
WB202	2020	Arcadia	Ampelakia	*Pica pica*	0	22,35486	37,38021
WB203	2020	Arcadia	Athinaion	*Pica pica*	0	22,24035	37,39669
WB204	2020	Arcadia	Athinaion	*Pica pica*	0	22,37553	37,40807
WB205	2020	Arcadia	Kandalos	*Pica pica*	0	22,26631	37,42798

WB206	2020	Arcadia	Arachamites	*Pica pica*	0	22,35805	37,61303
WB207	2020	Arcadia	Makri	*Pica pica*	0	22,33528	37,46493
WB208	2020	Arcadia	Lithovounia	*Pica pica*	0	22,40865	37,49620
WB209	2020	Arcadia	Neochori	*Pica pica*	0	22,38448	37,50046
WB210	2020	Arcadia	Tripoli	*Pica pica*	0	22,37399	37,49490
WB211	2020	Arcadia	Nestani	*Pica pica*	0	22,35483	37,50313
WB212	2020	Arcadia	Makri	*Turdus merula*	0	22,33231	37,46184
WB213	2020	Arcadia	Perivolia	*Corvus monedula*	0	22,16494	37,37957
WB214	2020	Arcadia	Megalopolis	*Corvus monendula*	1	22,16596	37,38540
WB215	2020	Arcadia	Merkovouni	*Garrulus glandarius*	0	22,37989	37,53723
WB216	2020	Arcadia	Vlachokerasia	*Garrulus glandarius*	0	22,35486	37,38021
WB217	2020	Arcadia	Tripoli	*Garrulus glandarius*	0	22,35483	37,50313
WB218	2020	Arcadia	Megalopolis	*Garrulus glandarius*	0	22,12596	37,40609
WB219	2020	Arcadia	Tripoli	*Garrulus glandarius*	1	22,41781	37,50735
WB220	2020	Arcadia	Paparis	*Garrulus glandarius*	0	22,25768	37,37093
WB221	2020	Arcadia	Tripoli	*Parus major*	0	22,36839	37,53110
WB222	2020	Arcadia	Tripoli	*Parus major*	0	22,36819	37,53113
WB223	2020	Arcadia	Thana	*Parus major*	0	22,36140	37,47445
WB224	2020	Arcadia	Rizes	*Passer domesticus*	0	22,46695	37,43924
WB225	2020	Arcadia	Asea	*Passer domesticus*	0	22,29021	37,40460
WB226	2020	Arcadia	Vervena	*Passer domesticus*	0	22,73675	37,43474
WB227	2020	Arcadia	Tripoli	*Passer domesticus*	0	22,41530	37,52437
WB228	2020	Arcadia	Tripoli	*Phylloscopus collybita*	0	22,34728	37,47757
WB229	2020	Arcadia	Tripoli	*Phylloscopus collybita*	0	22,41441	37,52421
WB230	2020	Arcadia	Nestani	*Pica pica*	0	22,46532	37,61943
WB231	2020	Arcadia	Loukas	*Pica pica*	0	22,40677	37,59155
WB232	2020	Arcadia	Kapsa	*Pica pica*	0	22,35805	37,61303
WB233	2020	Arcadia	Thana	*Pica pica*	0	22,36736	37,47067
WB234	2020	Arcadia	Ampelakia	*Pica pica*	0	22,28546	37,38454
WB235	2020	Arcadia	Megalopolis	*Pica pica*	0	22,14525	37,39412
WB236	2020	Arcadia	Tripoli	*Pica pica*	0	22,28103	37,38503
WB237	2020	Arcadia	Tripoli	*Pica pica*	0	22,28413	37,39806
WB238	2020	Arcadia	Steno	*Pica pica*	0	22,46340	37,49285
WB239	2020	Arcadia	Asea	*Pica pica*	1	22,27892	37,38905
WB240	2020	Arcadia	Astros	*Pica pica*	0	22,69200	37,41400
WB241	2020	Arcadia	Pelagos	*Pica pica*	0	22,42667	37,52058
WB242	2020	Arcadia	Tripoli	*Pica pica*	1	22,41502	37,50942
WB243	2020	Arcadia	Tripoli	*Pica pica*	1	22,26011	37,38603
WB244	2020	Arcadia	Tripoli	*Pica pica*	0	22,27931	37,35693
WB245	2020	Arcadia	Tripoli	*Pica pica*	0	22,33672	37,46628
WB246	2020	Arcadia	Tripoli	*Pica pica*	0	22,33025	37,46787
WB247	2020	Arcadia	Tripoli	*Pica pica*	0	22,66943	37,51235
WB248	2020	Arcadia	Tripoli	*Pica pica*	0	22,38506	37,50168
WB249	2020	Arcadia	Tripoli	*Pica pica*	0	22,41441	37,52421
WB250	2020	Arcadia	Monastiraki	*Pica pica*	0	22,75251	37,70680
WB251	2020	Arcadia	Monastiraki	*Pica pica*	0	22,75471	37,70573
WB252	2020	Arcadia	Agios Vasileios	*Pica pica*	0	22,42067	37,51089
WB253	2020	Arcadia	Dafni	*Pica pica*	0	22,27657	37,36417
WB254	2020	Arcadia	Athinaion	*Pica pica*	0	22,24317	37,40195
WB255	2020	Arcadia	Doriza	*Pica pica*	0	22,29800	37,43963
WB256	2020	Arcadia	Makri	*Pica pica*	0	22,32934	37,46919
WB257	2020	Arcadia	Agios Konstantinos	*Pica pica*	0	22,39322	37,52041
WB258	2020	Arcadia	Tripolis	*Curruca communis*	0	22,41521	37,59541
WB259	2020	Korinthia	Agios Ioannis	*Pica pica*	0	22,96537	37,77751

WB260	2020	Korinthia	Athikia	*Pica pica*	0	22,97966	37,86447
WB261	2020	Korinthia	Galataki	*Pica pica*	0	22,89012	37,87865
WB262	2020	Korinthia	Athikia	*Pica pica*	0	22,97966	37,86447
WB263	2020	Korinthia	Galataki	*Pica pica*	0	22,92800	37,81394
WB264	2020	Korinthia	Galataki	*Pica pica*	0	22,92800	37,81394
WB265	2020	Korinthia	Zeugolatio	*Pica pica*	0	22,78766	37,93915
WB266	2020	Korinthia	Xilokeriza	*Pica pica*	0	22,98200	37,89300
WB267	2020	Korinthia	Ancient Korinthos	*Pica pica*	0	22,86484	37,90396
WB268	2020	Korinthia	Examilia	*Pica pica*	0	22,92214	37,89460
WB269	2020	Korinthia	Korinthos	*Pica pica*	0	22,93335	37,92938
WB270	2020	Korinthia	Poulitsa	*Pica pica*	0	22,67839	38,00800
WB271	2020	Korinthia	Korinthos	*Passer domesticus*	0	22,93335	37,92938
WB272	2020	Korinthia	Athikia	*Passer domesticus*	0	22,92800	37,81394
WB273	2020	Korinthia	Korinthos	*Passer domesticus*	1	22,93441	37,92790
WB274	2020	Korinthia	Korinthos	*Passer domesticus*	0	22,93310	37,92930
WB275	2020	Korinthia	Korinthos	*Passer domesticus*	0	22,93441	37,92790
WB276	2020	Korinthia	Examilia	*Passer domesticus*	0	22,92214	37,89460
WB277	2020	Korinthia	Examilia	*Passer domesticus*	0	22,92214	37,89460
WB278	2020	Korinthia	Examilia	*Passer domesticus*	0	22,92214	37,89460
WB279	2020	Korinthia	Korinthos	*Passer domesticus*	1	22,93307	37,92936
WB280	2020	Korinthia	Korinthos	*Passer domesticus*	0	22,93310	37,92930
WB281	2020	Korinthia	Korinthos	*Passer domesticus*	0	22,93310	37,92930
WB282	2020	Korinthia	Korinthos	*Passer domesticus*	0	22,93310	37,92930
WB283	2020	Korinthia	Korinthos	*Passer domesticus*	0	22,93310	37,92930
WB284	2020	Korinthia	Korinthos	*Passer domesticus*	0	22,93310	37,92930
WB285	2020	Korinthia	Korinthos	*Passer domesticus*	0	22,93310	37,92930
WB286	2020	Korinthia	Korinthos	*Passer domesticus*	0	22,93310	37,92930
WB287	2020	Korinthia	Korinthos	*Passer domesticus*	0	22,93310	37,92930
WB288	2020	Korinthia	Korinthos	*Passer domesticus*	0	22,93310	37,92930
WB289	2020	Korinthia	Korinthos	*Passer domesticus*	0	22,93310	37,92930
WB290	2020	Korinthia	Korinthos	*Passer domesticus*	0	22,93310	37,92930
WB291	2020	Korinthia	Solomos	*Passer domesticus*	0	22,93335	37,92938
WB292	2020	Korinthia	Solomos	*Passer domesticus*	0	22,93335	37,92938
WB293	2020	Korinthia	Loutraki-Petrachora	*Pica pica*	0	22,98923	37,96188
WB294	2020	Korinthia	Examilia	*Pica pica*	0	22,92214	37,89460
WB295	2020	Korinthia	Galataki	*Pica pica*	0	22,92800	37,81394
WB296	2020	Korinthia	Galataki	*Pica pica*	0	22,92800	37,81394
WB297	2020	Korinthia	Sikiona	*Pica pica*	0	22,94580	37,91896
WB298	2020	Korinthia	Korinthos	*Pica pica*	1	22,95046	37,91252
WB299	2020	Korinthia	Korinthos	*Pica pica*	0	22,90774	37,92876
WB300	2020	Korinthia	Korinthos	*Pica pica*	0	22,92293	37,92853
WB301	2020	Korinthia	Korinthos	*Pica pica*	0	22,97202	37,92113
WB302	2020	Korinthia	Korinthos	*Pica pica*	0	22,93000	37,91023
WB303	2020	Korinthia	Korinthos	*Pica pica*	0	22,94344	37,92568
WB304	2020	Korinthia	Korinthos	*Pica pica*	0	22,91322	37,91080
WB305	2020	Korinthia	Kiato	*Pica pica*	1	22,73286	38,00008
WB306	2020	Korinthia	Kiato	*Pica pica*	0	22,74068	38,00601
WB307	2020	Korinthia	Korinthos	*Pica pica*	1	22,93441	37,92790
WB308	2020	Korinthia	Korinthos	*Pica pica*	1	22,93310	37,92930
WB309	2020	Korinthia	Korinthos	*Pica pica*	0	22,93307	37,92936
WB310	2020	Korinthia	Korinthos	*Pica pica*	0	22,93572	37,92973
WB311	2020	Korinthia	Korinthos	*Pica pica*	1	22,93335	37,92938

WB312	2020	Korinthia	Korinthos	*Pica pica*	0	22,93335	37,92938
WB313	2020	Korinthia	Solomos	*Pica pica*	1	22,89139	37,87025
WB314	2020	Korinthia	Korinthos	*Pica pica*	0	22,89582	37,87923
WB315	2020	Korinthia	Solomos	*Pica pica*	1	22,89575	37,87998
WB316	2020	Korinthia	Solomos	*Pica pica*	1	22,88349	37,88479
WB317	2020	Korinthia	Solomos	*Pica pica*	0	22,89012	37,87865
WB318	2020	Korinthia	Solomos	*Pica pica*	0	22,88533	37,87756
WB319	2020	Korinthia	Solomos	*Pica pica*	0	22,89298	37,88124
WB320	2020	Korinthia	Solomos	*Pica pica*	0	22,88715	37,87627
WB321	2020	Korinthia	Solomos	*Pica pica*	0	22,89299	37,88020
WB322	2020	Korinthia	Solomos	*Pica pica*	1	22,88349	37,88479
WB323	2020	Korinthia	Galataki	*Pica pica*	0	22,89012	37,87865
WB324	2020	Korinthia	Solomos	*Pica pica*	0	22,89176	37,87979
WB325	2020	Korinthia	Examilia	*Pica pica*	0	22,92214	37,89460
WB326	2020	Korinthia	Perigiali	*Pica pica*	0	22,83519	37,94442
WB327	2020	Korinthia	Korinthos	*Pica pica*	0	22,95046	37,91188
WB328	2020	Korinthia	Korinthos	*Pica pica*	0	22,94850	37,91299
WB329	2020	Korinthia	Sikiona	*Pica pica*	0	22,94580	37,91896
WB330	2020	Korinthia	Solomos	*Pica pica*	0	22,93335	37,92938
WB331	2020	Korinthia	Korinthos	*Pica pica*	0	22,93586	37,92952
WB332	2020	Korinthia	Korinthos	*Pica pica*	0	22,94880	37,92483
WB333	2020	Korinthia	Korinthos	*Streptopelia decaocto*	0	22,94989	37,92579
WB334	2020	Korinthia	Korinthos	*Streptopelia decaocto*	0	22,95431	37,91133
WB335	2020	Korinthia	Nemea	*Garrulus glandarius*	0	22,89900	37,92426
WB336	2020	Korinthia	Spathovouni	*Larus michahellis*	0	22,89904	37,92402
WB337	2020	Korinthia	Chiliomodi	*Parus major*	1	22,92766	37,92531
WB338	2020	Korinthia	Korinthos	*Passer domesticus*	0	22,92579	37,92528
WB339	2020	Korinthia	Korinthos	*Passer domesticus*	0	22,92591	37,92611
WB340	2020	Korinthia	Korinthos	*Passer domesticus*	0	22,95600	37,91700
WB341	2020	Korinthia	Korinthos	*Passer domesticus*	0	22,94900	37,92600
WB342	2020	Korinthia	Korinthos	*Pica pica*	0	22,93334	37,92949
WB343	2020	Korinthia	Korinthos	*Pica pica*	0	22,93537	37,92949
WB344	2020	Korinthia	Ancient Korinthos	*Pica pica*	0	22,94685	37,91297
WB345	2020	Korinthia	Xilokeriza	*Pica pica*	0	22,93397	37,92757
WB346	2020	Korinthia	Korinthos	*Pica pica*	0	22,93376	37,92711
WB347	2020	Korinthia	Korinthos	*Pica pica*	0	22,93511	37,92939
WB348	2020	Korinthia	Lechaio	*Pica pica*	0	22,85308	37,92634
WB349	2020	Korinthia	Examilia	*Pica pica*	1	22,94888	37,91258
WB350	2020	Korinthia	Solomos	*Pica pica*	1	22,89298	37,88124
WB351	2020	Korinthia	Solomos	*Pica pica*	1	22,89298	37,88124
WB352	2020	Korinthia	Chiliomodi	*Pica pica*	0	22,86849	37,79490
WB353	2020	Korinthia	Korinthos	*Pica pica*	0	22,95431	37,91133
WB354	2020	Korinthia	Korinthos	*Pica pica*	0	22,94344	37,92568
WB355	2020	Korinthia	Korinthos	*Pica pica*	0	22,91322	37,91080
WB356	2020	Korinthia	Korinthos	*Pica pica*	0	22,91524	37,91208
WB357	2020	Korinthia	Korinthos	*Pica pica*	0	22,91765	37,90722
WB358	2020	Korinthia	Korinthos	*Pica pica*	0	22,91732	37,90675
WB359	2020	Korinthia	Korinthos	*Pica pica*	0	22,91804	37,90657
WB360	2020	Korinthia	Korinthos	*Pica pica*	0	22,95431	37,91133
WB361	2020	Korinthia	Zeugolatio	*Turdus merula*	0	22,80513	37,94452
WB362	2020	Lakonia	Pakia	*Pica pica*	0	22,83737	36,74970
WB363	2020	Lakonia	Leimonas	*Pica pica*	0	22,68227	36,82029
WB364	2020	Lakonia	Stefania	*Pica pica*	0	22,59412	36,83994
WB365	2020	Lakonia	Gouves	*Pica pica*	0	22,74810	36,84441

WB366	2020	Lakonia	Gouves	*Pica pica*	0	22,76038	36,85512
WB367	2020	Lakonia	Koupia	*Pica pica*	0	22,90766	36,86226
WB368	2020	Lakonia	Peristeri	*Pica pica*	0	22,68339	36,86584
WB369	2020	Lakonia	Grammousa	*Pica pica*	0	22,66107	36,90332
WB370	2020	Lakonia	Kalyvia Sochas	*Pica pica*	0	22,41113	37,02368
WB371	2020	Lakonia	Afisia	*Pica pica*	0	22,02507	37,10463
WB372	2020	Lakonia	Vasara	*Pica pica*	0	22,00799	37,17366
WB373	2020	Lakonia	Papadianika	*Turdus merula*	0	22,87084	36,71124
WB374	2020	Lakonia	Voutianoi	*Turdus philomelos*	0	22,05800	37,15423
WB375	2020	Lakonia	Sparti	*Corvus cornix*	0	22,43219	37,05340
WB376	2020	Lakonia	Xirokampi	*Garrulus glandarius*	0	22,47277	37,03849
WB377	2020	Lakonia	Sparti	*Garrulus glandarius*	0	22,47277	37,03849
WB378	2020	Lakonia	Molaoi	*Garrulus glandarius*	0	22,85899	36,81650
WB379	2020	Lakonia	Skala	*Garrulus glandarius*	0	22,87512	36,80010
WB380	2020	Lakonia	Elos	*Passer domesticus*	0	22,70247	36,83123
WB381	2020	Lakonia	Elos	*Passer domesticus*	0	22,70312	36,83065
WB382	2020	Lakonia	Elos	*Passer domesticus*	1	22,70232	36,83131
WB383	2020	Lakonia	Elos	*Passer domesticus*	1	22,70274	36,82913
WB384	2020	Lakonia	Elos	*Passer domesticus*	0	22,69310	36,83479
WB385	2020	Lakonia	Skala	*Passer domesticus*	0	22,70803	36,83339
WB386	2020	Lakonia	Skala	*Passer domesticus*	0	22,70804	36,83287
WB387	2020	Lakonia	Skala	*Passer domesticus*	0	22,70122	36,83781
WB388	2020	Lakonia	Skala	*Passer domesticus*	0	22,70128	36,82911
WB389	2020	Lakonia	Skala	*Passer domesticus*	0	22,70236	36,82937
WB390	2020	Lakonia	Skala	*Passer domesticus*	1	22,67490	36,85456
WB391	2020	Lakonia	Elos	*Passer domesticus*	0	22,70128	36,82911
WB392	2020	Lakonia	Elos	*Passer domesticus*	0	22,70236	36,82937
WB393	2020	Lakonia	Elos	*Passer domesticus*	0	22,70284	36,83669
WB394	2020	Lakonia	Skala	*Passer domesticus*	0	22,67163	36,85772
WB395	2020	Lakonia	Elos	*Passer domesticus*	0	22,67462	36,85775
WB396	2020	Lakonia	Magoulas	*Pica pica*	0	22,41416	37,08370
WB397	2020	Lakonia	Stefania	*Pica pica*	0	22,60735	36,84681
WB398	2020	Lakonia	Apidea	*Pica pica*	0	22,79128	36,88550
WB399	2020	Lakonia	Vlachioti	*Pica pica*	0	22,70940	36,87114
WB400	2020	Lakonia	Glykovryshi	*Pica pica*	0	22,78831	36,79460
WB401	2020	Lakonia	Geraki	*Pica pica*	0	22,69571	36,99285
WB402	2020	Lakonia	Amykles	*Pica pica*	0	22,43538	37,02115
WB403	2020	Lakonia	Gythio	*Pica pica*	0	22,52166	36,78198
WB404	2020	Lakonia	Chosiari	*Pica pica*	0	22,50253	36,72104
WB405	2020	Lakonia	Dafni	*Pica pica*	0	22,53198	36,93077
WB406	2020	Lakonia	Skala	*Pica pica*	0	22,67924	36,84685
WB407	2020	Lakonia	Asopos	*Pica pica*	0	22,67462	36,85775
WB408	2020	Lakonia	Agios Ioannis	*Pica pica*	0	22,39525	37,05520
WB409	2020	Lakonia	Skouras	*Pica pica*	0	22,49589	37,01932
WB410	2020	Lakonia	Molaoi	*Pica pica*	0	22,87512	36,80010
WB411	2020	Lakonia	Molaoi	*Pica pica*	1	22,94726	36,76736
WB412	2020	Lakonia	Molaoi	*Pica pica*	0	22,86131	36,79523
WB413	2020	Lakonia	Sykea	*Pica pica*	1	22,93870	36,76979
WB414	2020	Lakonia	Neapoli	*Pica pica*	0	23,06700	36,50700
WB415	2020	Lakonia	Elos	*Pica pica*	0	22,69310	36,83479
WB416	2020	Lakonia	Aphishia	*Pica pica*	0	22,70803	36,83339
WB417	2020	Lakonia	Neapoli	*Pica pica*	0	22,70804	36,83287
WB418	2020	Lakonia	Sykea	*Pica pica*	0	22,70122	36,83781

WB419	2020	Lakonia	Elos	*Pica pica*	0	22,70128	36,82911
WB420	2020	Lakonia	Amykles	*Pica pica*	0	22,70236	36,82937
WB421	2020	Lakonia	Sparti	*Turdus merula*	0	22,70284	36,83669
WB422	2020	Lakonia	Elos	*Turdus merula*	0	22,70128	36,82911
WB423	2020	Lakonia	Elos	*Turdus merula*	0	22,69310	36,83479
WB424	2020	Lakonia	Elos	*Turdus merula*	0	22,70247	36,83123
WB425	2020	Lakonia	Elos	*Turdus merula*	0	22,70247	36,83123
WB426	2020	Messinia	Pyrgos	*Buteo buteo*	0	22,45465	37,08512
WB427	2020	Messinia	Loggas	*Pica pica*	0	21,90189	36,85229
WB428	2020	Messinia	Verga	*Pica pica*	0	22,18118	36,98490
WB429	2020	Messinia	Verga	*Pica pica*	0	22,17264	37,01315
WB430	2020	Messinia	Messini	*Pica pica*	0	22,15913	36,99922
WB431	2020	Messinia	Messini	*Pica pica*	0	21,71041	37,06474
WB432	2020	Messinia	Aris	*Pica pica*	0	22,00331	37,10420
WB433	2020	Messinia	Androusa	*Pica pica*	0	21,96531	37,12895
WB434	2020	Messinia	Aristodimio	*Pica pica*	0	22,42452	37,15096
WB435	2020	Messinia	Arfara	*Pica pica*	0	22,01800	37,16200
WB436	2020	Messinia	Skala	*Pica pica*	0	22,04600	37,15000
WB437	2020	Messinia	Mauromati Ithomis	*Pica pica*	0	21,94214	37,21350
WB438	2020	Messinia	Neochori Ithomis	*Pica pica*	0	22,31555	37,35970
WB439	2020	Messinia	Chora	*Turdus merula*	0	21,70919	37,07934
WB440	2020	Messinia	Kalamata	*Apus apus*	0	22,13146	37,02987
WB441	2020	Messinia	Gargalianoi	*Bubo bubo*	0	21,69244	37,09285
WB442	2020	Messinia	Gargalianoi	*Bubo bubo*	0	21,65937	37,11366
WB443	2020	Messinia	Gargalianoi	*Columba livia*	0	21,62851	37,06173
WB444	2020	Messinia	Kalamata	*Columba livia*	0	22,08786	37,04549
WB445	2020	Messinia	Kallithea	*Corvus cornix*	0	21,79756	36,87606
WB446	2020	Messinia	Aristomenis	*Garrulus glandarius*	0	21,83224	37,09349
WB447	2020	Messinia	Chora	*Hirundo rustica*	0	21,72131	37,05400
WB448	2020	Messinia	Avia	*Parus major*	1	22,14504	36,93336
WB449	2020	Messinia	Avia	*Parus major*	0	22,14859	36,94511
WB450	2020	Messinia	Messini	*Parus major*	1	22,03204	37,06804
WB451	2020	Messinia	Avia	*Parus major*	0	22,03248	37,06805
WB452	2020	Messinia	Valira	*Passer domesticus*	0	21,98368	37,15647
WB453	2020	Messinia	Valira	*Passer domesticus*	0	21,98549	37,15684
WB454	2020	Messinia	Valira	*Passer domesticus*	0	21,98509	37,15703
WB455	2020	Messinia	Valira	*Passer domesticus*	0	21,98037	37,15604
WB456	2020	Messinia	Arfara	*Passer domesticus*	0	21,98518	37,15704
WB457	2020	Messinia	Valira	*Passer domesticus*	0	21,98518	37,15704
WB458	2020	Messinia	Valira	*Passer domesticus*	0	21,98400	37,15700
WB459	2020	Messinia	Loggas	*Passer domesticus*	0	21,90407	36,86897
WB460	2020	Messinia	Valira	*Passer domesticus*	0	21,98400	37,15700
WB461	2020	Messinia	Filiatra	*Passer domesticus*	0	21,57766	37,15906
WB462	2020	Messinia	Filiatra	*Passer domesticus*	0	21,59315	37,16116
WB463	2020	Messinia	Kalamata	*Passer hispaniolensis*	1	22,04387	37,05189
WB464	2020	Messinia	Kalamata	*Passer hispaniolensis*	1	22,06571	37,04948
WB465	2020	Messinia	Mikromani	*Passer hispaniolensis*	0	22,07960	37,05256
WB466	2020	Messinia	Kalamata	*Passer montanus*	0	22,03194	37,06763
WB467	2020	Messinia	Mikromani	*Passer montanus*	0	22,02034	37,08728
WB468	2020	Messinia	Mikromani	*Passer montanus*	0	22,01886	37,08686
WB469	2020	Messinia	Mikromani	*Passer montanus*	0	22,03454	37,08181
WB470	2020	Messinia	Messini	*Phylloscopus collybita*	0	21,98981	37,05461

WB471	2020	Messinia	Verga	*Pica pica*	0	22,15913	36,99922
WB472	2020	Messinia	Avia	*Pica pica*	0	22,15148	36,96640
WB473	2020	Messinia	Konstantinoi	*Pica pica*	0	21,94457	37,29043
WB474	2020	Messinia	Meligalas	*Pica pica*	0	21,96911	37,21778
WB475	2020	Messinia	Madeni	*Pica pica*	0	21,96040	37,03961
WB476	2020	Messinia	Filiatra	*Pica pica*	0	21,60372	37,15501
WB477	2020	Messinia	Kyparissia	*Pica pica*	0	21,65666	37,23346
WB478	2020	Messinia	Verga	*Pica pica*	0	22,15769	36,99786
WB479	2020	Messinia	Kopanaki	*Pica pica*	0	21,81824	37,29500
WB480	2020	Messinia	Gargalianoi	*Pica pica*	0	21,64257	37,06133
WB481	2020	Messinia	Valira	*Pica pica*	1	21,98439	37,15707
WB482	2020	Messinia	Loggas	*Pica pica*	0	21,90487	36,87373
WB483	2020	Messinia	Antikalamos	*Pica pica*	0	22,06589	37,06778
WB484	2020	Messinia	Piperitsa	*Pica pica*	0	22,00034	37,07810
WB485	2020	Messinia	Gargalianoi	*Pica pica*	0	21,64257	37,06133
WB486	2020	Messinia	Arios	*Pica pica*	0	21,94457	37,11825
WB487	2020	Messinia	Messini	*Curruca communis*	1	22,03248	37,06805
WB488	2020	Messinia	Messini	*Turdus merula*	0	22,00960	37,03581
WB489	2020	Messinia	Kalamata	*Turdus merula*	0	22,06571	37,04948
WB490	2020	Messinia	Pilos	*Ardea purpurea*	1	21,67659	36,97247
WB491	2020	Messinia	Gargalianoi	*Athene noctua*	1	21,63248	37,06854
WB492	2020	Messinia	Alonia	*Curruca melanocephala*	0	21,99436	37,13176
WB493	2020	Messinia	Filiatra	*Srix aluco*	1	21,58128	37,18062

0; negative PCR result, 1; positive PCR result

Table S3. Collection sites of mosquito pools

Number of Trap	Regional Unit	Trap Fixed Location	Environment	Number of positive mosquito pools	WGS84_X	WGS84_Y
1	Lakonia	Asteri	Natural Wetland	0	22,730213	36,82435
2	Lakonia	Leimonas	Agricultural	0	22,681031	36,82216
3	Lakonia	Trinisa	Natural Wetland	1	22,613164	36,81997
4	Lakonia	Skala	Natural Wetland / Agricultural	1	22,660495	36,85241
5	Lakonia	St. George	Natural Wetland / Agricultural	1	22,67698	36,85411
6	Lakonia	Leimonas	Natural Wetland / Agricultural	0	22,679508	36,82514
7	Lakonia	Elos	Residential	0	22,696484	36,83294
8	Lakonia	Elos	Agricultural	1	22,697697	36,82916
9	Argolida	Timenio	Natural Wetland	1	22,730008	37,59112
10	Argolida	Monastiraki	Agricultural	0	22,747912	37,70375
11	Argolida	Laloukas	Agricultural	2	22,783149	37,62889
12	Argolida	Koutsopodi	Residential	0	22,709225	37,68324
13	Argolida	Nafplio	Natural Wetland	0	22,799845	37,58422
14	Argolida	Fixti	Agricultural	2	22,722568	37,72408
15	Argolida	Dalamanara	Agricultural	1	22,768875	37,62468
16	Argolida	Nea Tirintha	Agricultural	1	22,819047	37,6109
17	Argolida	Era	Agricultural	0	22,760489	37,65435
18	Argolida	Anyphi	Agricultural	0	22,782189	37,66084
19	Korinthia	Korinthos	Residential	0	22,933776	37,93555
20	Korinthia	Ancient Korinthos	Agricultural	1	22,875762	37,9032
21	Korinthia	Vrachai	Agricultural	0	22,804221	37,95528
22	Korinthia	Solomos	Agricultural	0	22,887904	37,87415
23	Korinthia	Chiliomodi	Agricultural	1	22,871798	37,81501
24	Korinthia	Lechaio	Agricultural	2	22,841537	37,92017
25	Messinia	Kalamata	Natural Wetland / Agricultural	0	22,064313	37,03398
26	Messinia	Asprochoma	Natural Wetland / Agricultural	0	22,072015	37,04786

27	Messinia	Mikromani	Agricultural	1	22,0296	37,07606
28	Messinia	Aris	Agricultural	0	22,004018	37,09883
29	Messinia	Messini Airport	Natural Wetland	0	22,017536	37,05759
30	Arcadia	Tripoli	Agricultural	3	22,384984	37,49745
31	Arcadia	St. Vasileios	Agricultural	1	22,425658	37,51151
32	Arcadia	Kato Vervena	Natural Wetland	0	22,747183	37,44126
33	Arcadia	Neochori	Natural Wetland / Agricultural	0	22,75071	37,37319
34	Arcadia	Paralio Astro	Residential	1	22,757428	37,4077
35	Arcadia	St. Andrews	Natural Wetland	1	22,7830835	37,37144

Table S4. Environmental variables.

Environmental variable	Code
Annual mean temperature (°C)	clima1
Maximum temperature of warmest month (°C)	clima5
Mean temperature of wettest quarter (°C)	clima8
Mean temperature of driest quarter (°C)	clima9
Mean temperature of warmest quarter (°C)	clima10
Mean temperature of coldest quarter (°C)	clima11
Total annual precipitation (mm)	clima12
Altitude (m)	dem
Distance from water collections (m)	waterdis
Livestock densities (sheep, goats, cattle/km^2)	sheepd
Land uses (44 classes)	landcorine
Human population density (people/km^2)	popden
April NDVI[1]	aprndvi
May NDVI	mayndvi

[1]NDVI: normalized difference vegetation index

Table S5. Categories of land uses.

Categories of Land Uses			Code
**1st Level**	**2nd Level**	**3rd Level**	
**Artificial surfaces**	Urban fabric	Continuous urban fabric	1
		Discontinuous urban fabric	2
	Industrial, commercial and transport units	Industrial or commercial units	3
		Road and rail networks and associated land	4
		Port areas	5
		Airports	6
	Mine, dump and construction sites	Mineral extraction sites	7
		Dump sites	8
		Construction sites	9
	Artificial, non-agricultural vegetated areas	Green urban areas	10
		Sport and leisure facilities	11
**Agricultural areas**	Arable land	Non-irrigated arable land	12
		Permanently irrigated land	13
		Rice fields	14
	Permanent crops	Vineyards	15
		Fruit trees and berry plantations	16
		Olive groves	17
	Pastures	Pastures	18
	Heterogeneous agricultural areas	Annual crops associated with permanent crops	19
		Complex cultivation patterns	20
		Land principally occupied by agriculture, with significant areas of natural vegetation	21
		Agro-forestry areas	22
**Forest and semi natural areas**	Forests	Broad-leaved forest	23
		Coniferous forest	24
		Mixed forest	25
	Scrub and/or herbaceous vegetation associations	Natural grasslands	26
		Moors and heathland	27
		Sclerophyllous vegetation	28
		Transitional woodland-shrub	29
	Open spaces with little or no vegetation	Beaches, dunes, sands	30
		Bare rocks	31
		Sparsely vegetated areas	32
		Burnt areas	33
		Glaciers and perpetual snow	34
**Wetlands**	Inland wetlands	Inland marshes	35
		Peat bogs	36
	Maritime wetlands	Salt marshes	37
		Salines	38
		Intertidal flats	39
**Water bodies**	Inland waters	Water courses	40
		Water bodies	41
	Marine waters	Coastal lagoons	42
		Estuaries	43
		Sea and ocean	44
